# Team Preceptorship Model: A Solution for Students' Clinical Experience

**DOI:** 10.5402/2011/530357

**Published:** 2011-05-04

**Authors:** Angela Cooper Brathwaite, Manon Lemonde

**Affiliations:** ^1^Lawrence Bloomberg Faculty of Nursing, University of Toronto, 155 College Street, Suite 130, Toronto, ON, Canada M5T 1P8; ^2^University of Ontario Institute of Technology, 200 Simcoe Street North, P.O. Box 385, Oshawa, ON, Canada L1H 7L7

## Abstract

There is a shortage of registered nurses in developed countries, and this shortage is due to the aging nursing workforce, demand for healthcare services, and shortage of nursing professors to teach students. In order to increase the number of clinical placements for nursing students, the authors developed and implemented a collaborative preceptorship model between a Canadian University and Public Health Department to facilitate the clinical experiences of Bachelor of Science of Nursing (BScN) students. This paper describes the Team Preceptorship Model which guided the clinical experience of nine students and 14 preceptors. It also highlights the model's evaluation, strengths, and limitations.

## 1. Introduction

Nursing is experiencing a significant shortage of registered nurses around the world, particularly in developed countries such as the United States and Canada [1–4]. This shortage is due to the increased demand for healthcare services, shortage of nursing professors, and the aging population of the nursing workforce. In Ontario, Canada, the shortage is intensified by the requirement for all registered nurses to be prepared at the Bachelor of Science of Nursing (BScN) level [5]. Most BScN programs include public health clinical experience as an intricate part of it. In order to meet this requirement, a Public Health Department (PHD) collaborated with a Faculty of Health Sciences to develop a collaborative preceptorship model called the Team Preceptorship Model (TPM), which was used to guide students' clinical experience in a public health setting. The purpose of this paper is to describe the strengths, limitations, and applicability of the TPM, which contributed to students' clinical experience and learning in Public Health Nursing.

Prior to reviewing the literature on collaborative preceptorship models, we examined the definitions and purposes of preceptorship to nursing education. Preceptorship is defined as a one-to-one relationship between a registered nurse (RN) and a nursing student during an intense, time-limited clinical experience to facilitate students' learning, and is supported by nursing faculty [6]. Preceptorship is important to nursing education for several reasons: it assists nursing students to incorporate theory into practice, integrates students into the practice setting within the organization, allows the student to apply learning and internalize the role and values of the profession within a nurturing and supportive relationship, and assists in recruiting nursing students into the profession [7]. This paper begins with a brief review of the literature, which is followed by a description of the collaborative development of the TPM, methods of evaluating the model and concludes with a description of the model's strength, limitations, and evaluation of it.

## 2. Review of the Literature

In the past decade, several investigators [8–13] reported positive findings of collaborative preceptor models between schools of nursing and service agencies. Results of these studies showed that nursing students had a positive experience, expanded their knowledge, increased their confidence, and integrated their skills with real-life situations. These benefits have been found across a range of preceptorship models that have been developed over time.

The Preceptor Model involving a single student being precepted by a single nurse originated in the time of Florence Nightingale and is commonly utilized today [12]. According to Nordgren et al. [12], the Preceptor Model is mainly used in North America for senior nursing students during their final term of study; where one nurse-preceptor is responsible for the clinical teaching of a single student and the faculty member is responsible for supervising the general experience of the student. Similarly, in the Integrated Clinical Preceptor Model [14], students participate in planning their clinical experience, the preceptor acts as the clinical teacher, role model, and mentor, and the faculty member is resource for both student and preceptor. Evaluation of these models revealed positive outcomes for students: increased confidence, acquisition of skills and experiential knowledge in a specific clinical specialty, and preparation for practice following graduation; preceptors increase their scope of service (research and career development); faculty increased their productivity in research and scholarship. However, students expressed negative effects of the Nordgren's model, such as lack of control over their experience and the need to be more assertive in expressing their learning needs to preceptors. Some students felt that preceptors were occasionally not as sensitive to their needs.

Another collaborative model was the Clinical Teaching Associate Model developed by Phillips and Kaempfer [15]. It constituted of one preceptor directing the clinical teaching of a group of students. Evaluation results showed positive outcomes for students and faculty such as a variety of patient care experiences for students, increased students' confidence, and faculty increased or freed-up time to address more complex issues. Alternatively, the Modified Clinical Teaching Model developed by Baird et al. [16] involved beginning and advance students being taught in small groups by one preceptor. The faculty member was always present in the clinical area during students' clinical experience and assisted the preceptor in planning students' learning experiences. Evaluation of this model revealed several benefits for students and faculty members such as, increased contact time between students and preceptors, better usage of faculty time, and instruction of students by clinical experts. A limitation of the aforementioned models was that fewer nursing students receive individual or 1 : 1 preceptor's support. In Phillips and Kaempfer's model [15], preceptors had difficulty in covering patients' assignments since preceptors were assigned according to students' needs and not unit needs.

Lastly, Happell [17] developed a collaborative model between a university and health care agency. This preceptorship model was based upon the preceptor-preceptee relationship and factors which influence clinical learning from the perspective of nursing students and clinicians who taught them. The main focus of the model was teaching and learning. In this model, the preceptor was the role model who inspired students to develop clinical skills and embrace the inherent value of nursing practice, respected the student as a member of the nursing team, and recognized them as inexperience and lacking confidence. Thus, the preceptor provided a supportive learning environment for students. The university and healthcare agency recognized their dual roles in the partnership and valued the preceptorship as an essential component of high-quality nursing education and provided resources to sustain it. A critical review of this model revealed several benefits for students and preceptors: students gained specialized clinical skills and received regular feedback on their performance in a positive working environment while preceptors contributed to the theoretical program and development of learning objectives for students and had designated time to preceptor students within their workload. The strength of this model was it applicability to other settings and clinical specialties.

In summary, five preceptorship models were reviewed in terms of process, outcomes, and limitations. Despite their differences in design and processes, evaluation of the models indicated positive clinical outcomes for students, preceptors, and faculty. A major limitation of these models was that they were used in acute care settings and none included attention to preceptorship in community placements like public health departments. Additionally, most of the models have not taken a staff team approach to facilitate students' clinical experiences. Such a shared preceptorship approach was developed in collaboration between PHD and a school of nursing that allowed students' placements in a public health setting. In order to meet this gap, a PHD collaborated with the Faculty of Health Sciences at a Canadian University to design and implement a TPM, which is described in subsequent paragraphs.

## 3. Team Preceptorship Model (TPM)

Team Preceptorship Model is an innovative model based on the premise of collaborative mentoring and is defined as nurses working together with several members of the partnership between the school and the PHD, to provide public health preceptored experiences for BScN students by mutually coaching and facilitating their personal and professional growth [18]. Each individual in the partnership is recognized for unique experiences, skills, and knowledge that he or she brings to the learning experience. Each person participates in a direct or supportive role as a member of the partnership, and each person coaches the student in “interactive, interpersonal processes that involve the acquisition of appropriate skills, actions, and abilities that form the basis of professional practice” Gearlish [19]. The TPM is defined by the authors as an integrated approach that fosters a reciprocal relationship amongst the student, preceptor, program staff, and faculty member to provide rich community experiences for undergraduate nursing students. 

In each program at the PHD, a group of preceptors work as a team. Smaller programs have a team of two preceptors with two students assigned to each preceptor, whereas the larger programs have a team of four preceptors with 2-3 students assigned to each preceptor. Additionally, the team of preceptors and their students are supported by program staff in their respective programs. This structure allows for flexibility, creativity, and support for students and preceptors, when selecting learning experiences for students. For example, a student may be assigned to a project or activity that a program staff is responsible for if this activity fits within the students' learning objectives. Consequently, the program staff provides coaching, mentoring, and feedback to the student as well as contributes to his or her evaluation. A detailed description of the model now follows.

The objectives of the TPM are to (1) significantly increase the number and proportion of BScN learners receiving public health nursing experiences, (2) create a learning environment in which the preceptor and learners feel supported (3) create an environment in which the learning responsibility is shared among all members of the team (preceptors, student placement coordinator, mentors, learners, managers, and faculty) (4) increase collaboration and communication between the University and PHD, and (5) prepare nursing students with competencies for public health practice. In order to meet these objectives, the TPM is comprised of three components which are described in subsequent paragraphs.

### 3.1. Student Orientation to PHD

Students attend a two-day PHD orientation program that helps to familiarize them with public health topics including policies and practices, organizational structure, services provided to the community, geographical location of communities that comprise the area of service, public health standards, nursing competencies, roles and responsibilities of the preceptors, students' responsibilities and expectations in the TPM partnership, health promotion theories, activities, and roles of the team members. During the first day of orientation, the PHD coordinator gives an overview of the PH Divisions (Nursing and Nutrition, Environmental Health, Oral Health Services, Emergency Medical Services, Administration, and Infant & Child Development). She also explains practical concerns of locations of offices, working hours, personal safety, and process for obtaining personal identification cards. The preceptor facilitates the second day of orientation, providing students with specific information regarding each program to which they are assigned.

### 3.2. Assignment of Students to Public Health Projects and Activities

Throughout the clinical placements, students are expected to take an active role within their assigned programs (Prevention of Injury and Substance Misuse, Chronic Disease, Infectious Disease Control and Prevention or Reproductive and Child Health) and are recognized and respected for their contributions as team members. Thus, the preceptors collaborate with program peers and manager to select appropriate activities and projects for each group of students. Criteria of appropriateness include ability to realistically implement projects during placement time frame and opportunity to learn the public health nursing role.

### 3.3. Mid-Term and End of Term Evaluation

Students' evaluation is the most explicit component involving the agency and faculty collaboration. An important aspect of students' learning experience is reflection which allows students to critically review and analyze their experiences and to examine alternative perspectives and solutions. The preceptor provides feedback and comments in an open, honest, and nonthreatening manner, contributing to a formative evaluation of the clinical experience. Effective feedback promotes professional and personal growth when it is provided in a respectful, supportive approach [6]. The preceptor provides mid-term and final evaluations to each student in collaboration with faculty advisors according to school requirements. These evaluations are an integral component of the practicum experience and are based on third year evaluation criteria/competencies, to determine students' progress and achievement of the learning objectives. 

### 3.4. Roles of Team Members

Each member of the team holds particular responsibilities in their given roles. Role of the preceptor is comprised of providing orientation to students in their respective programs; reviewing organizational policies and procedures and code of conduct; reviewing with students the reciprocal expectations for the preceptorship such as meeting times, role of students to contacting preceptors and providing feedback; coaching and role models; communicating effectively with all members of the team; and conducting mid-term and final evaluations for students.

The role of the students include attending University and agency orientation programs, reviewing expectations of placement, developing a learning plan related to the practice area in collaboration with the preceptor, maintaining open communication with preceptor and faculty advisor, and providing constructive feedback to the preceptor about the preceptorship experience and compiling with the academic expectations.

Similarly, the program staff provides support to preceptors and learners, provides feedback to learners, contributes to their evaluation, and assists learners in meeting their learning needs. Additionally, the program managers support the TPM model and its implementation within the respective programs. Finally, the student placement coordinator is responsible for recruiting preceptors and organizing the preceptors' and students' orientations, facilitating preceptor and student debriefing at mid-and end of term, and liaising between the PHD and University. Alternatively, the faculty's roles include communicating with student placement coordinator and preceptors, monitoring and evaluating students' progress and learning experiences, and determining the final grade.

## 4. Methods

### 4.1. Evaluating the TPM

We received ethical approval from the involved organizations' Ethical Review Boards (University and PHD) prior to recruiting participants. Data were collected using a focus group format. An interview guide was created to help in facilitating the two focus groups (one for students and one for preceptors). These groups took place at 12 weeks of the placement. Nine students and 14 preceptors participated in the respective groups. A focus group format was useful for this project because it assisted in information recall and produced rich data [20]. Additionally, students were comfortable in sharing with one another and PHNs reflected together about the preceptorship role, to generate deeper descriptions of their experiences.

A faculty member moderated the discussions with the preceptor group, while a manager at the PHD facilitated the discussions with the student group to ensure noncoercion in data collection and dependability of data analysis. Neither the faculty member nor the manager was associated with the clinical experience of students or recruiting of participants. At the beginning of each group, the facilitators introduced themselves and had members of the groups introduce themselves to one another. This was followed by them conducting the discussions using the interview guide. Data from the focus groups were audio taped and then transcribed by a research assistant (RA) from the university. Specific interview questions included (1) based on your experience, what do you feel are the advantages of the team preceptorship model? and (2) what do you feel are the disadvantages of the team preceptorship model? Participants were also asked to share their thoughts as they reflected on the whole clinical experience through these open-ended questions.

The researchers used Hsueg and Shannon methods [21] of content analysis to analyze the data to identify themes with supportive quotes. The principal researcher played a predominant role in data analysis. She facilitated internal consistency in the interpretation of the data by assuming the major responsibility for conducting the data analysis, making interpretative notes, and communicating with other team members as the data analysis proceeded. Then, the other researcher independently reviewed the transcripts and themes as well as made interpretative notes. Finally, the team of researchers met to discuss and verify consistency in interpretation of the data and reached consensus on the final themes.

The researchers confirmed credibility of the findings by having participants review the themes and corresponding quotes to see whether they recognize the findings of the study to be true to their expectations. Participants confirmed that the themes and quotes were accurate. Thus, members' checking confirmed the accuracy of the findings [22, 23].

## 5. Results

In the preceptor focus group, three themes emerged from the discussion of question 1 and one theme from question 2. Each theme was described and summarized with direct quotes from participants to provide rich description of the themes. They were support for preceptors and students, good communication among team members, and collaboration among team members. The theme from question 2 was feeling overworked. These themes with supporting quotes are in Table 1. The student focus group was asked the same questions regarding the advantages and disadvantages of the TPM. Two themes emerged from the discussion in question 1. They were accessibility of preceptors and preceptors' expertise. One theme emerged from question 2. It was that public health activities took too long to be implemented. These themes with corresponding quotes are in Table 2.

## 6. Discussion

The TPM model was evaluated to determine its applicability and relevance to a public health setting. In the evaluation of the model, several strengths were identified: accessibility of preceptors to students, preceptors' expertise, support for preceptors and students, good communication among team members, and collaboration among team members. These positive results were found by other researchers [18, 19, 24–28]. For example, Myrick reported that preceptors' accessibility and expertise contributed in promoting students' critical thinking and broadened their clinical knowledge and expertise. Similarly, Ferguson [18] reported that faculty's support to preceptors facilitated their ability to evaluate students' performance, while Usher et al. [24] and Yonge et al. [25] emphasized support from the clinical agency and fellow workers were vital to the preceptor's role. Hyrkas and Shoemaker [29] also found a positive association between preceptors' perceptions of support and commitment to their role. Additionally, Latham and colleagues [26] purported that partnership among frontline RNs improved the culture of support and healthy workplace environment.

Limitations of the model were that preceptors felt overworked and students felt that public health activities took too long to be implemented. Pertaining to preceptors' feelings of being overworked, Coates and Gormley [27] and Yonge et al. [25] found that the biggest barrier to effective preceptoring of students was preceptors' lack of time with students. Preceptors had acknowledged that being assigned to students meant taking extra time and effort to teach them, which consequently increased their workloads. This finding corroborated with Leners et al. [30] who indicated that preceptors' workload was a major disadvantage and huge concern for preceptors. It is important to note that each preceptor was given one day per week of dedicated time funded by the PHD to support students within the PH setting for the term. The dedicated time was used for coaching and mentoring students' activities, attending meetings with the Student Placement Coordinator and faculty, as well as performing students' evaluations.

 Alternatively, students' perception of public health is accurate, in that it takes time and effort to implement social change [9]. This perception of public health is not a limitation of the model but reveals the challenges students face when learning and implementing public health strategies to enhance the health of a population. Lastly, results of this evaluation will be used to improve the TPM. It has clinical utility and can be adapted into other settings to increase the volume of nursing students in clinical placements. For example, the number of students' clinical placements in the PHD had increased from 14 to 35 per term during the TPM implementation. Special attention should be given to students' learning during orientation to explain the length of time it takes to implement social changes in the community or society.

### 6.1. Implications for Practice

Preceptors and students faced different challenges during implementation of the TPM. Preceptors felt overworked and students perceived that public health activities took too long to be implemented. These results have implications for clinical practice. Firstly, administrative personnel at the PHD should reduce preceptors' workload to accommodate the preceptorship role, that means, delegating some of their assigned activities to peers and increasing the dedicated time for preceptoring students. The dedicated time should be increased to one and a half days per week for the first four weeks of the clinical placement and then reduced to one day per week for the remaining eight weeks of the academic term. Increasing the time and reducing their workloads will help to relieve their stress as well as the potential for burnout. Additionally, a larger pool of preceptors should be trained and rotated every second academic year, thus preventing preceptors' burnout. 

On the other hand, the placement coordinator and preceptors should include theories of change such as Prochaska's 5-stages of change: precontemplation, contemplation, preparation, action, and maintenance [31] and Lewin's change model: unfreeze/refreeze [32] into students' orientation. The Placement coordinator should explain and apply these models to public health practice in order to increase students' understanding of the complexity and time needed to plan and implement interventions for the population. Kumm et al.'s article [31] goes beyond Prochaska's five stages of change by introducing “motivational techniques” that allow a practitioner to match his/her approach with a client or colleague to the change that the person is experiencing. Similarly, the preceptor can utilize “motivational interviewing” to assist students in identifying the stage of change where they are at, by applying Prochaska's 5-stages of change to their present situation. They may also discuss participatory program planning [33], which would assist students in understanding the different types of stakeholders who collaborate with public health professionals in successfully developing and implementing health promotion interventions for the population.

## 7. Conclusion

This paper highlights components of the TPM model, its strengths, limitations, and implications for clinical practice. The TPM is an innovative approach to teach a large group of students in a public health department. Evaluation results are consistent with principles of the TPM, which embraced collaboration among all partners and was a crucial part of the model's functioning. Based on evaluation results, it seems appropriate to move forward with permanently utilizing this model for future placements of students.

## Figures and Tables

**Table 1:** Themes from preceptors' group.

Themes	Quotes
Support for preceptors and students	R1 “For us, one of the advantages was rather than having to look for a whole bunch of opportunities for the students, one set of our team looked for activities that were specific to breast feeding. Another part of the team looked for home visits, and another part looked for any prenatal opportunities. And then we opened that to all the students. So you didn't have to do all the work. It was good”.
	R2 “I appreciated having a manager oversee what the preceptors do. So each preceptor did not have to deal with preceptor type issues, program activities, students' questions and stuff like that. It was nice just to have it be more streamlined”.
	R4 “…I think because there were more than one student, they worked as a team as well to do whatever their assignment was”.

Collaboration among team members	R3 “We didn't feel like the onus of responsibility was just on the preceptors. It was there, more working groups and students were mentoring one another as well. So that really helped to alleviate the pressure of the day to day activities. Whereas our involvement was more around the learning plans, evaluation and that type of thing. So that really worked much better this time. We coordinated much better this time with more preceptors and developing activities for the students”.

Good communication among team members	R3 “…it worked better this time. The last time we had a problem with staff coverage. And everything has flowed much easier this time. Communication is really open because we have three circles (of nursing staff) in the Child Health Program. Most of it to cover client services and the students have opportunity to discuss public health practices and interventions”.

Feeling overworked	R6 “… the struggle is that you feel constantly overworked. So the crazy question is how this semester experience is different from the last year. The amount of time that I spent was huge, writing those evaluations and communication tools to give the faculty in order to evaluate the students”.

**Table 2:** Themes from students' group.

Themes	Quotes
Accessibility of preceptors	R1 “Well I think it was good to know that your preceptor was always there when you had a question or you didn't know what to do. We had our own cubicles which were pretty close to our preceptors, so we knew where to find her if we had any questions and if we needed anything”.
	R5 “…and also sometimes we had one on one with her because a preceptor tends to have 2 or 3 students while the faculty could have 10 or more students. So it was easier to get hold of them and they spent more time with you”.

Expertise of preceptors	R3 “It was really nice because they have the expertise in this area, if we were… with a faculty, who has a background in public health, it would make a difference. We benefitted more from preceptors' expertise and knowledge than from faculty”.
	R6 “Another thing about this semester, lots of people was like me. We got a lot of opportunities to do activities outside the Health Department with our preceptors. They really showed us what else they do in the community, and included us and made sure that we knew how our project was incorporated within the community”.
	R7 “You identify in your learning plan that your preceptor is there to highlight learning experiences for you that correspond with your learning plan, to help you accomplish your goals. For me, it was a very effective approach. I mean we get a lot more experience by having a knowledgeable preceptor with much more diverse experience as opposed to having one faculty member with 10 to 12 students. We got much attention”.

Public health activities took too long to be implemented	R4 “Community nursing (home health) is very different from public health nursing. I think because of the differences we were kind of impatient, banging our heads”.
	R9 “I could not do anything because things take such a long time with meeting after meeting and they have teams, so no decision could be made unless they met all together. And so it was a very slow progress. Coming from a hospital where things happen overnight, there was not an end to any of the projects that Reproductive Health was working on. I got a chance to experience a home visit which was fabulous and that was probably the best afternoon of the entire 13 weeks”.
